# Si and Ge based metallic core/shell nanowires for nano-electronic device applications

**DOI:** 10.1038/s41598-018-35225-6

**Published:** 2018-11-15

**Authors:** Prabal Dev Bhuyan, Ashok Kumar, Yogesh Sonvane, P. N. Gajjar, Rita Magri, Sanjeev K. Gupta

**Affiliations:** 1 Computational Materials and Nanoscience Group, Department of Physics and Electronics, St. Xavier’s College, Ahmedabad, 380009 India; 2 Department of Physical Sciences, School of Basic and Applied Sciences, Central University of Punjab, Bathinda, Punjab 151001 India; 3 Department of Applied Physics, S.V. National Institute of Technology, Surat, 395007 India; 4 Department of Physics, Gujarat University, Ahmedabad, 380009 India; 5 Department of Physics, Informatics and Mathematics (FIM), University of Modena and Reggio Emilia, Via Campi 213/A, Modena, Italy

## Abstract

One dimensional heterostructure nanowires (NWs) have attracted a large attention due to the possibility of easily tuning their energy gap, a useful property for application to next generation electronic devices. In this work, we propose new core/shell NW systems where Ge and Si shells are built around very thin As and Sb cores. The modification in the electronic properties arises due to the induced compressive strain experienced by the metal core region which is attributed to the lattice-mismatch with the shell region. As/Ge and As/Si nanowires undergo a semiconducting-to-metal transition on increasing the diameter of the shell. The current-voltage (I-V) characteristics of the nanowires show a negative differential conductance (NDC) effect for small diameters that could lead to their application in atomic scale device(s) for fast switching. In addition, an ohmic behavior and upto 300% increment of the current value is achieved on just doubling the shell region. The resistivity of nanowires decreases with the increase in diameter. These characteristics make these NWs suitable candidates for application as electron connectors in nanoelectronic devices.

## Introduction

Since the synthesis of carbon nanotubes (CNTs) in the beginning of 1990s^1^, one dimensional (1D) nanostructures have attracted a huge interest in the last three decades. CNTs were believed to be among the most important building blocks for the next generation electronic devices. After decades of progress in nanotechnology, both theoretically and experimentally, nanowires have opened up substantial opportunities for the development of nanoelectronic and optoelectronic devices. Therefore, several studies have demonstrated the application of nanowires (NWs) in the field of electron devices^2–9^, logic gates^2,10,11^, optoelectronics^12–14^, photonics^15,16^, chemical and biological sensing devices^10,17–20^, non-volatile memories^21^ etc. Likewise, 1D heterostructure NWs have been extensively studied for next-generation device applications^22,23^.

Recently, extensive efforts have been devoted to grow and study core/shell NWs consisting of semiconductor/semiconductor^24,25^, semiconductor/metal^26^, metal/metal oxide^27,28^, metal oxide/metal oxide^29–32^, metal/metal^33,34^ heterostructure(s). Particularly, semiconductor core/shell NWs such as SiGe, have gained much attention due to their remarkable transport and optical properties^22^ which are attributed to the spatial localization of electrons and holes. The electronic properties of these NWs have a strong dependence on their size, shape and chemical composition^35^. For example, Amato *et al*. have shown that the geometry and composition of Si/Ge core/shell NWs strongly influence their thermodynamic stability and electronic properties. Peng *et al*.^36^ have shown that the intrinsic strain between Ge and Si layers is responsible for the reduction of the band gap in Si/Ge core/shell NWs from their pristine counterparts. In addition, external strain can be used to tune the band gap of the NWs, and promote a direct-to-indirect (or vice-versa) band gap transition. It has also been reported that Si/Ge NW quantum dot FET shows a superior performance over the Si nanowire FET due to a gate field induced switching mechanism^37^. Recently, synthesized Si/SiC core/shell NWs paved the way to biocompatible nanowire based sensors due to the chemically inertness and hydrophilic surface of SiC^38^. This semiconducting core/shell NWs have obtained enormous applications in the field of FET, IC circuits, logic gates, solar cells in nanoelectronic devices^22,37,39,40^. The versatile electronic, transport and optical properties of core/shell NWs which depend on the configuration and composition of the core and shell of the NWs have motivated us to go beyond IV-IV and III-V nanowire systems and study the electronic and transport properties of core/shell NWs with a different composition.

Both Sb and As have gained a very high interest as 2D materials, known as antimonene and arsenene. The puckered structure of antimonene and arsenene has shown dynamical stability^41,42^ and possesses an indirect bandgap. Experimentally, monolayers and nanoribbons of these structures have been successfully synthesized^43,44^. Antimonene has also shown possible applications in microelectronics and optoelectronics nanodevices and solar cell applications^45^. Similarly, it has been reported that arsenene could find applications as photodiodes, light emitting diodes and in the field of solar cells^42^. Moreover, GeSb-NWs of diameter 40–100 nm have been achieved experimentally and applied in memory devices^46^. In addition multi-layered and few-layered GeAs has also been grown in the laboratory and studied for applications in optical devices^23,47^. The synthesized nanowires with these combinations motivate us to study their core/shell structural properties. Growth of pure As and Sb nanowires was reported^48^. Thus, it is very possible that also (GeSi)/(AsSb) core-shell nanowires can be synthesized depositing Si or Ge after the growth of the pure metallic nanowires and adapting the growth conditions to avoid specie intermixing.

In the light of these results, it can be interesting to study the electronic structure of Sb and As when the dimension is reduced from 2D to 1D and, further, when the systems are subjected to the additional confinement due to the presence of the shell. Thus, our work considers Si and Ge based nanowire geometries with a As and Sb core that can very well be fabricated in the near future.

In this work, we have focused on Si or Ge based metallic As- and Sb- core/shell nanowires and studied their morphology and transport properties. Finding the appropriate strain imposed on the core, we can tune the metallic properties of the NWs. Diameter dependent IV- characteristics are also simulated in order to provide a picture as comprehensive as possible. We have investigated the suitability of these nanowires as electron connectors for nano-electronic devices and other technological applications.

## Model and Methodology

Geometry relaxations and electronic structure calculations are performed by using the SIESTA simulation package^49^. We have used numerical atomic orbitals basis with double zeta polarization (DZP) throughout the calculations. Troullier Martin norm conserving pseudopotential is used to treat the electron ion interactions^50^. The exchange and correlation energies for electron-electron interactions are treated within the generalized gradient approximation using Perdew- Burke-Ernzerhof (PBE) parametrization^51^. A vacuum of 20 Å along the x and y directions is considered to ensure that no interactions occur between the neighboring nanowire’s images. The NWs are optimized until the forces on each atom are lower than 0.01 eV/Å by using a conjugate gradients (CG) method. The Brillouin zone is sampled with a 1 × 1 × 12 Monkhorst Pack grid. We have calculated the bands in 30 K-points along the Γ-Z direction for the electronic band structure calculation.

We have considered two different cores of arsenic (As) and antimony (Sb), which are wrapped by 1 and 2 monolayers (ML) of germanium (Ge) and silicon (Si). The dangling bonds on the surface of the NWs are saturated by hydrogen atoms. For the Sb_core_/Ge_shell_ NW, the core consists of 6 atoms for a diameter 3.64 Å. The NW, wrapped by 1 ML has 40 atoms (6 Sb atoms, 18 Ge atoms and 16 H atoms) in a unit cell with a diameter 14.61 Å (1.46 nm). The thickness of the NW is increased to 22.79 Å (2.28 nm) with 78 atoms (6 Sb atoms, 48 Ge atoms and 24 H atoms) per unit cell when 2Si/Ge MLs wrap the As/Sb cores. Techniques for the growth of NWs with always smaller radii are constantly being devised and the fabrication of tiny NWs has been reported by a number of researchers. Christopher Koenigsmann *et al*. have synthesized experimentally Pd/Pt core/shell nanowires with a diameter of 2.0 ± 0.5 nm and shown their application as catalysts for oxygen reduction reactions^52^. Recently, ReS_2_-NW with 0.65 nm diameter has been synthesized experimentally by using a chemical vapor deposition process^53^. Furthermore, K. B. Dhungana *et al*. have studied ultrathin boron nanowire structure with diameter 1.5–1.7 Å. They have reported the dynamical stability of 1D boron NW(s) from phonon analysis of the structures^37^.

**Figure 1:**
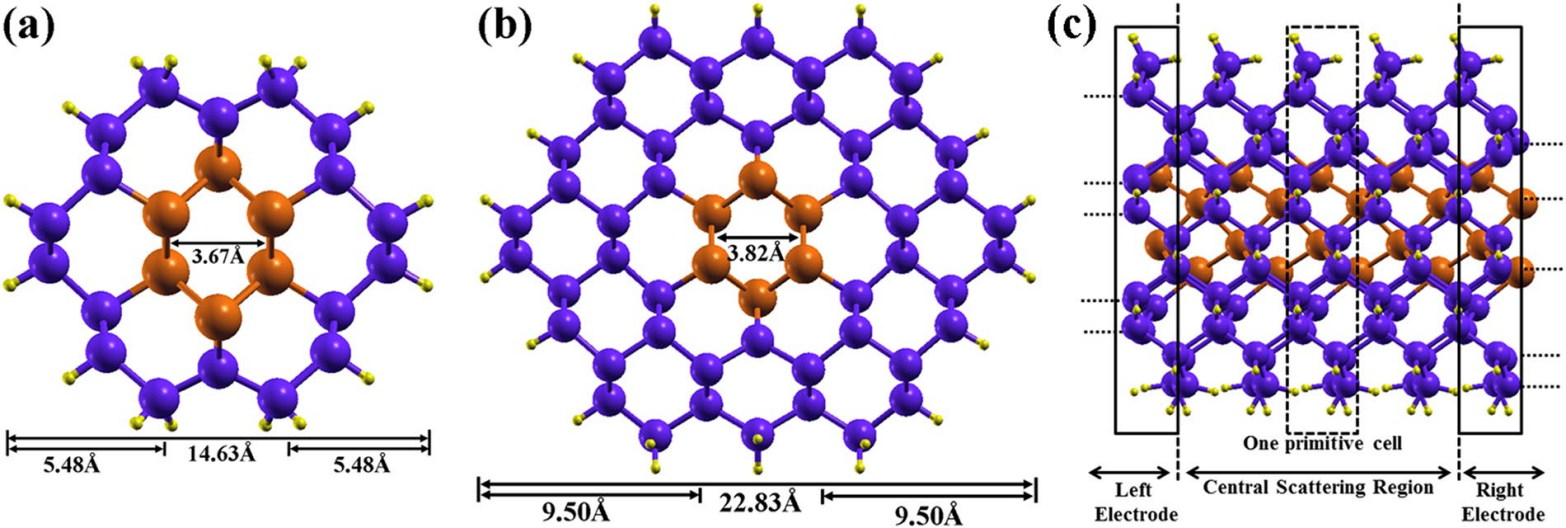
(**a**) and (**b**) Front views of the optimized structures of Sb/Ge core shell nanowires. (**a**) Sb/Ge NW with Sb core, wrapped by 1 ML Ge-shell, denoted Sb/Ge (R). (**b**) Sb-core wrapped by 2 ML Ge-shell having the total diameter of 22.83 Å, denoted Sb/Ge (2 R). Similar structures have been considered for As/Ge, As/Si and Sb/Si. (**c**) Schematic view of the two-probes core/shell NW system having 5 primitive cells along the z-axis. The central scattering region, left electrode and right electrode are indicated.

The electronic transport properties are studied by the non-equilibrium Green’s function (NEGF) techniques, within the Keldysh formalism, based on the density functional theory as implemented in the TranSIESTA module within the SIESTA package^54^. A two probe system is designed to study the transport properties of the considered core/shell nanowire confined in a central scattering region (SR) in contact with semi-infinite left electrode (LE) and right electrode (RE) as shown in Fig. 1(c). We have considered both the electrodes and the SR made of the same material. The total length of the central scattering region is 12 Å, which consists of three primitive cells. The current passing through the scattering region is calculated by using the Landauer-Buttiker formula^55^,

1 $$I({V}_{b})={G}_{0}{\int }_{{\mu }_{R}}^{{\mu }_{L}}T(E,{V}_{b})dE$$

where, G_0_ = 2(e^2^/h) is the unit of quantum conductance and T(E, V_bias_) is the transmission probability of an electron incident at an energy E through the device under the bias voltage V_bias_. The bias voltage between the two electrodes of different electrochemical potential μ_L_(left) and μ_R_ (right) is given by *eV*_*bias*_ = *μ*_*L*_ − *μ*_*R*_. We have increased the applied bias voltage (V_b_) in steps of 0.1 V. The solution of the converged density matrix is used as the initial input for the Green’s functions in the self-consistent cycle of the next step.

## Results and Discussions

### Structural Properties

First, we have optimized the pure As-, Sb-, Ge- and Si- NWs having 40 atoms and the axial parameters are calculated to be 4.96 Å, 4.35 Å, 4.17 Å and 3.92 Å, respectively. The pristine nanowires of Ge, Sb and Si show a semiconducting behavior, while the As-NW shows metallic behavior. We show the electronic band structures of pure Sb and As nanowires (ESI, Fig. S1). The band gaps of Ge, Sb and Si NWs are calculated to be 1.50 eV, 0.09 eV and 1.80 eV, respectively. The pristine Sb-NW, which shows a band gap of 0.09 eV, undergoes a transition from semi-metallic to metallic behavior when the NW is wrapped by 1 ML (monolayer) -Ge and -Si shell. On the contrary, the pristine As-NW shows a metallic behaviour and undergoes a transition from metallic to semi-metallic behavior when the NW is wrapped by 1 ML -Ge and -Si shell. The lattice constants of the core/shell nanowires with different diameters are smaller than their pristine NW counterparts. It is found that both the core and shell components of the NWs experience a compressive strain in all the considered NWs except for the Sb/Si (R) and As/Ge (2 R) NWs, due to the lattice mismatch at the interface (Table 1).

**Table 1:** Equilibrium lattice parameters and equivalent strain experienced by the cores and the shells for the considered core/shell NWs.

Core/shell NW	Axial lattice constant, a_z_ (Å)	Strain to core (%)	Strain to shell (%)
As/Ge (R)	4.12	−12.3	−1.30
As/Si (R)	3.91	−16.6	−0.35
Sb/Ge (R)	4.16	−4.52	−0.22
Sb/Si (R)	4.03	−7.46	+2.68
As/Ge (2 R)	4.18	−10.8	+0.34
As/Si (2 R)	3.90	−16.8	−0.60
Sb/Ge (2 R)	4.13	−5.14	−0.87
Sb/Si (2 R)	3.91	−10.26	−0.43

### Electronic Transport Properties

**Figure 2:**
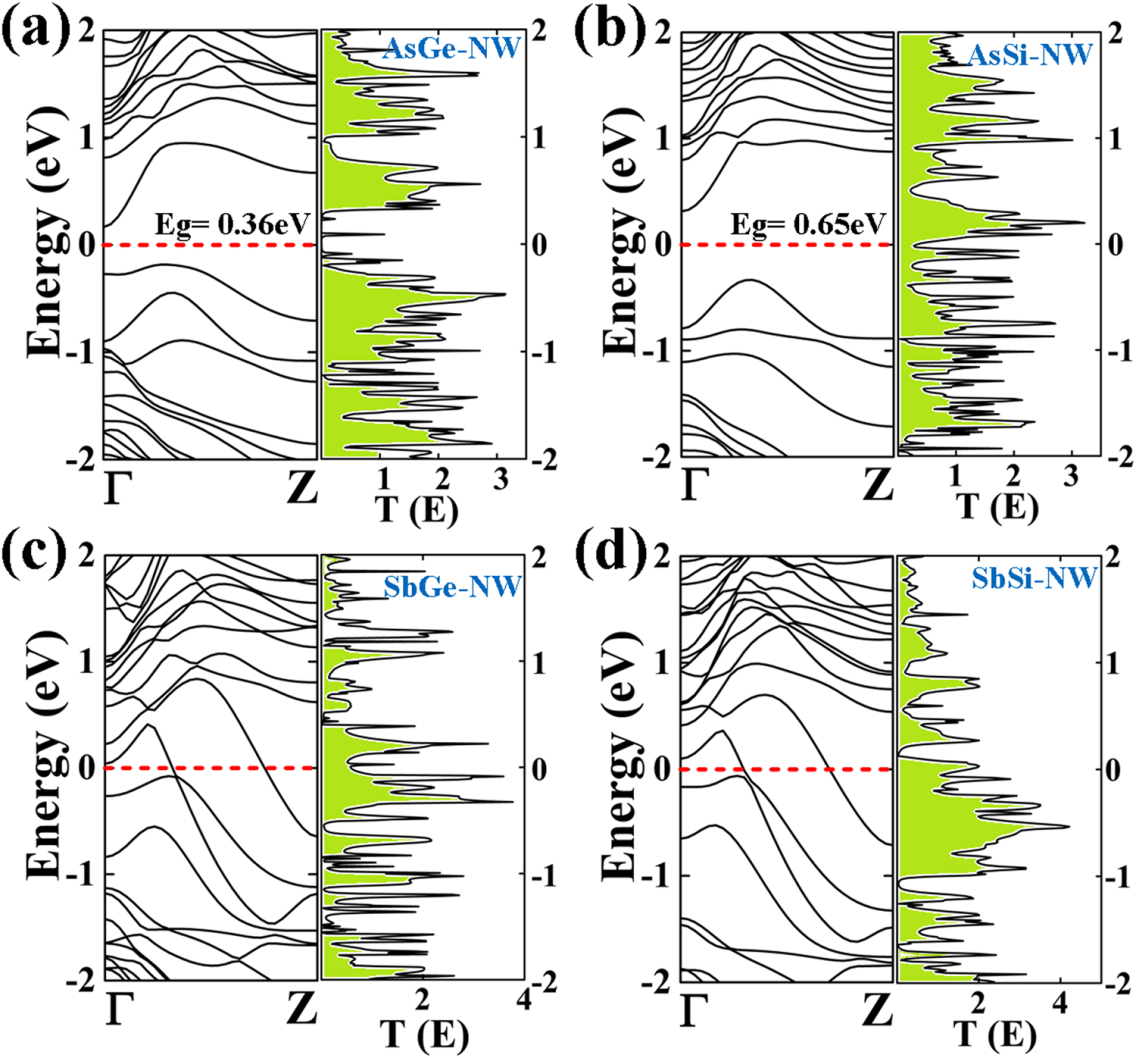
Electronic band structures of (**a**) As/Ge (R) (**b**) As/Si (R) (**c**) Sb/Ge (R) and (**d**) Sb/Si (R) core/shell nanowires. The corresponding transmission spectrum as function of energy (eV) under zero bias voltage of each NW is shown on the right side.

**Figure 3:**
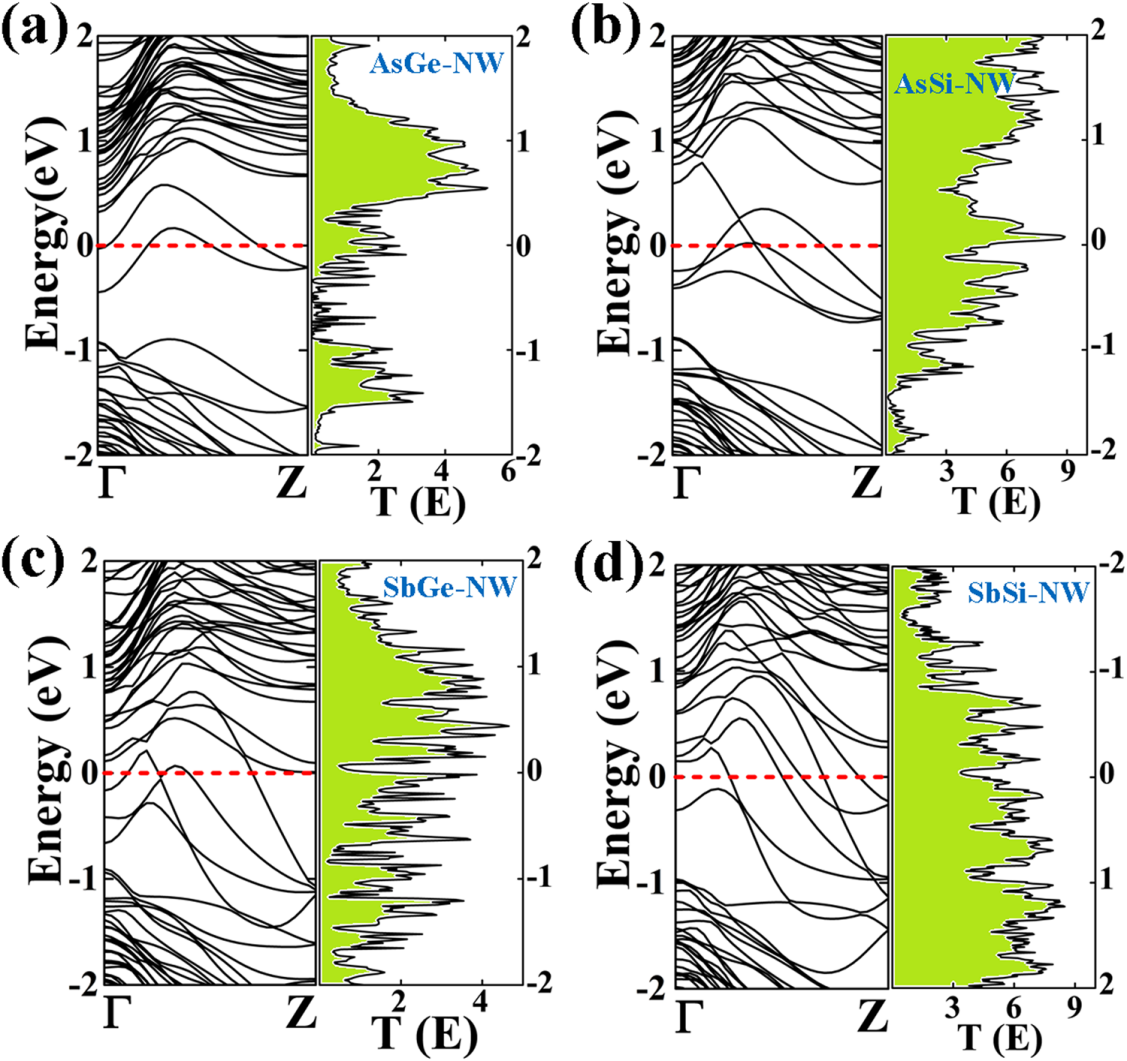
Electronic band structures of (**a**) As/Ge (2 R) (**b**) As/Si (2 R) (**c**) Sb/Ge (2 R) and (**d**) Sb/Si (2 R) core/shell nanowires. The corresponding transmission spectrum as a function of energy (eV) under zero bias voltage of each NW is shown on the right side.

In Figs 2 and 3, the band structures of the As/Ge, As/Si, Sb/Ge and Sb/Si core/shell NWs with different diameters are shown. The As/Ge (R) and As/Si (R) NWs are semiconducting with indirect band gaps 0.36 eV and 0.65 eV, respectively. However, both the nanowires undergo a semiconducting to metal transition on increasing the diameters (Fig. 3(a,b)). The As core wrapped with 2 ML Ge or Si shows a quantum ballistic conductance of 3G_0_ and 4G_0_, respectively. Note, that the band lines crossing the Fermi level can be attributed to quantum conductance in the units of G_0_ = 2e^2^/h, where, e is the electron charge and h is the Planck’s constant^56^. The quantum conductance gives information about the electron transport in the NW without electron-electron or electron-phonon scattering. The nanowires with the Sb-core and diameter R show metallic behavior with a quantum conductance of 2G_0_ which increases to 4G_0_ for Sb/Ge and 5G_0_ for Sb/Si NWs.

The calculated zero-bias transmission spectrum is shown with the corresponding band structure. The spike peaks in the spectrum are related to the available conductance channels due to the presence of bands. Sb/Ge (R) and Sb/Si (R) NWs show 2G_0_ conductance at the Fermi energy that are consistent with the electronic band structure since two band lines are crossing the Fermi level. The As/Ge (R)-NW shows zero conductance at the Fermi level, which is in accordance with a band gap energy in its band structure. In the case of As/Si (R)-NW, zero conductance without a Van Hove singularity is consistent with the corresponding semiconducting nature of the band structure.

The conductance values of the 2 R NWs from the transmission spectra are also consistent with the corresponding band structures (Figs 2 and 3). In order to get a further insight into the electronic properties of the considered core-shell nanowires, the orbital contribution near the Fermi level is extracted by calculating the partial density of states (PDOS) for all the core/shell NWs (ESI, Figs S2–S5). It is found that both valence and conduction bands around the Fermi energy are mainly dominated by the As-p orbitals for the As/Ge (R) and As/Si (R) NWs. A minor contributions from the shell Si and Ge p orbitals are also present around the Fermi level, whereas for all the NWs structures, the valence and conduction bands are totally dominated by the Si-p or Ge-p orbitals. The metallic behavior of the Sb/Ge and Sb/Si NWs is due to the contribution of the Sb-p orbitals. It has also observed that the contribution of the As-p orbitals is also large at the Fermi level for the As/Ge and As/Si NWs of higher diameter. Similarly, for Sb/Ge and Sb/Si NWs, the Fermi level is dominated by a core contribution.

**Figure 4:**
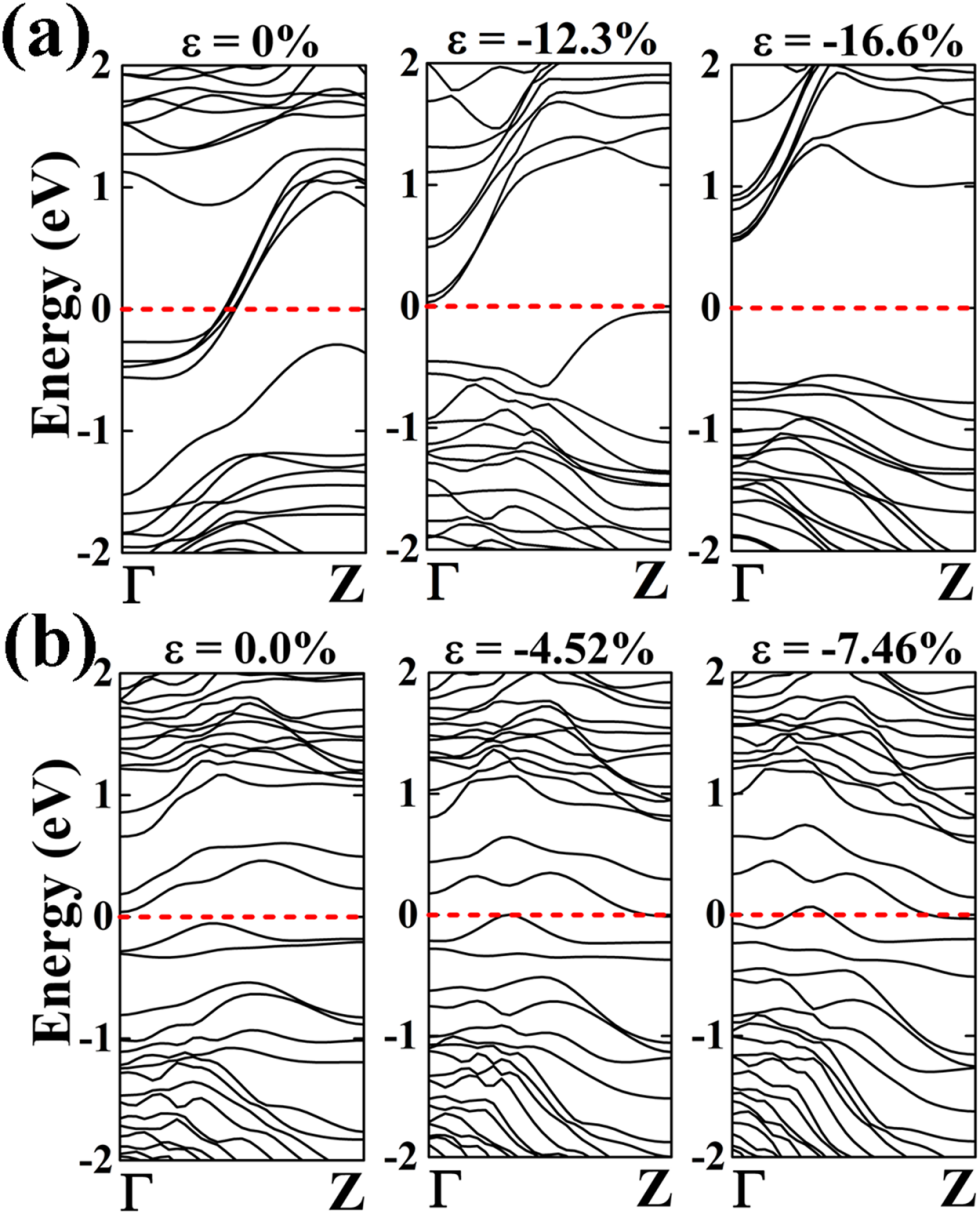
Band diagrams of (**a**) As and (**b**) Sb NW with diameter R at different degrees of strain (Ɛ). (**a**) The unstrained band structure of As, the band structure at Ɛ = −12.3% corresponding to the strain induced by the 1ML-Ge-shell, and at Ɛ = −16.6% corresponding to the strain induced by the 1 ML-Si-shell are shown. Similarly, in (**b**) the unstrained band structure of Sb, the band structure at Ɛ = −4.52% corresponding to the strain induced by the 1 ML-Ge-shell, and at Ɛ = −7.46% corresponding to the strain induced by the 1 ML-Si-shell are shown.

In order to get a deeper insight into the electronic behavior of the NWs, we have considered the application of an external mechanical strain to pristine As and Sb NWs. Note that the core part experiences an intrinsic strain in the given core/shell NW, e.g., the As core experiences a 12.3% (16.6%) compressive strain when the core is wrapped by 1 ML Ge-shell (Si-shell). Figure 4(a) presents the electronic structure of pristine As-NW as a function of uniaxial strain. Interestingly, the opening of a band gap is induced by applying a mechanical strain. However, the calculated energy gap of pure As-NW at 12.3% strain is smaller than the gap of the As/Ge-NW. This could be due to the neglecting of the strain experienced by the Ge-shell. The energy gap of the As-NW widens up at 16.6% strain and shows consistency with that of the As/Si NW. The Sb-core, wrapped by 1 ML Ge-shell experiences an intrinsic 4.25% strain, while the core wrapped by 1 ML Si-shell experiences an intrinsic 7.46% strain. The band structure of the pure Sb-NW is presented in Fig. 4(b) as a function of the uniaxial strain. The pure unstrained Sb-NW behaves as a semiconducting material with an indirect band gap of 0.09 eV. However it undergoes a semiconducting-to-metallic transition at 4.52% compressive strain. The conduction band minimum (CBM) moves down to the Fermi level and crosses it at Z point while the valence band maximum (VBM) crosses the Fermi energy along the Γ-Z direction. The Sb-NW at 4.52% strain shows a quantum conductance of 2G_0_ which is in accordance with the result obtained for the Sb/Ge core/shell NW. A similar behavior is also observed for the Sb-NW at 7.46% strain. The VBM moves up and crosses the Fermi energy level at two different points along the Γ-Z direction hence enhancing the quantum conductance to 2G_0_. This analysis shows that the change in the band structures and electronic transport properties are closely related to the intrinsic strain experienced by the core/shell NWs. Note that Amato *et al*. have also observed metallic behavior in Si/SiC core/shell NWs at a given diameter due to the intrinsic strain induced on the core region^38^.

We have observed that the As/Si and As/Ge nanowire undergoes a semiconducting-to-metallic transition as the diameter increases from 1.1 nm to 2.0 nm with no significant difference in the strain to core. Looking at their band structures, we notice that the main structures in the valence and conduction bands are still very similar, only the smaller peak which is below the Fermi energy in the thinner shell case moves up across the Fermi energy as the shell thickness doubles. This behavior cannot be explained only in terms of the strain acting on the NW metallic core. The PDOS (ESI, Figs S2–S5) of these nanowires show that to this structure contribute also the p states of the Si shell. Thus, the shifting of the peak to higher energies and at the Fermi level, which causes the transition from a semiconducting to a metallic behavior, shows that the strain to the shell and possibly the hybridization between the p states of the metal in the core and the p states of the semiconductor in the shell across the NW interface play a role in the band structure of these NWs.

Thus, the Si and Ge shells of different thicknesses lead to a tuning of the electronic structure of the As and Sb nanowires mostly through the strain to the core, but also through a state hybridization between the metal and semiconductor (also strained) orbitals at the interface.

### Current-Voltage Characteristics

**Figure 5:**
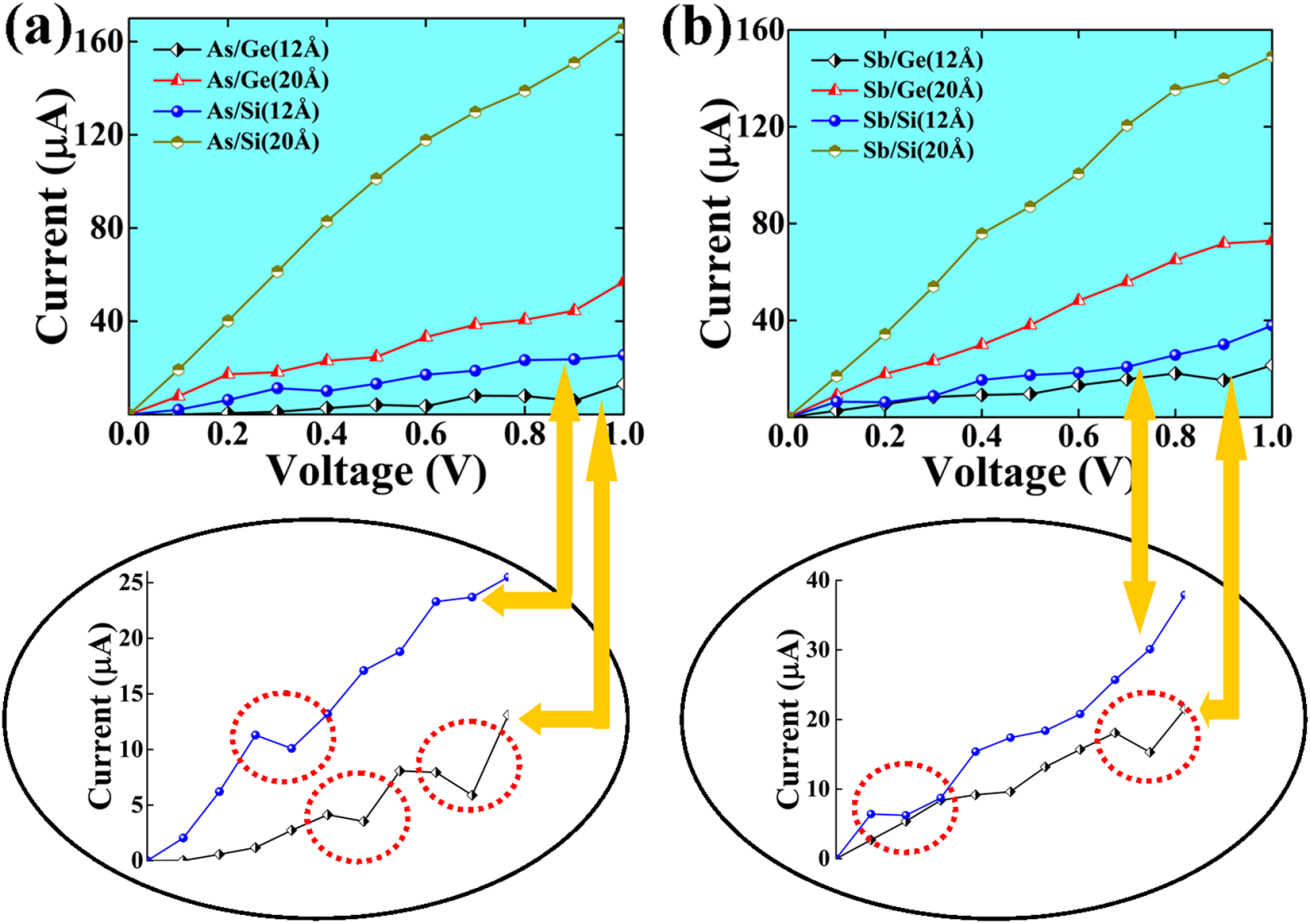
Current-Voltage (I-V) plot for various core/shell NWs with different diameters for bias voltages from 0 to 1.0 V. In the inset, the NDC regions are indicated.

Next, we have studied the current-voltage (IV) characteristics for the given core/shell NWs by employing the NEGF technique. Density functional theory (DFT) together with NEGF formulation to calculate I-V characteristics is a ground state theory at absolute zero, which offer very useful and reliable information of material properties at 0 K^57^. The applied voltage shifts the Fermi level of the left electrode with respect to the Fermi level of the right electrode. Once the energy of the top of the valence band of the left electrode matches with the bottom of the conduction band of the right electrode; the current start flowing through the scattering region. The current-voltage characteristics for the core/shell nanowires are presented in Fig. 5. We observe that the core wrapped by the Si-shell shows a larger conductivity as compared to the NWs with a Ge-shell. It is well known that in the ballistic regime the mobility depends on the effective masses of carriers and line stiffness of nanowires. Effective masses relate to the band dispersion at the critical points and they are different in the nanowires from the bulk materials. Furthermore, it has been shown in Table 1 that the strain to the core region is more for Si shell NWs as compared to Ge shell NWs. This strain may change the dispersion of the electronic bands edges and line stiffness of core/shell NWs which results into a change in the carrier mobility and hence in the magnitude of the current through the scattering region. Therefore, the cores experiencing a higher strain by the Si-shell give rise to a much higher conductivity.

**Figure 6:**
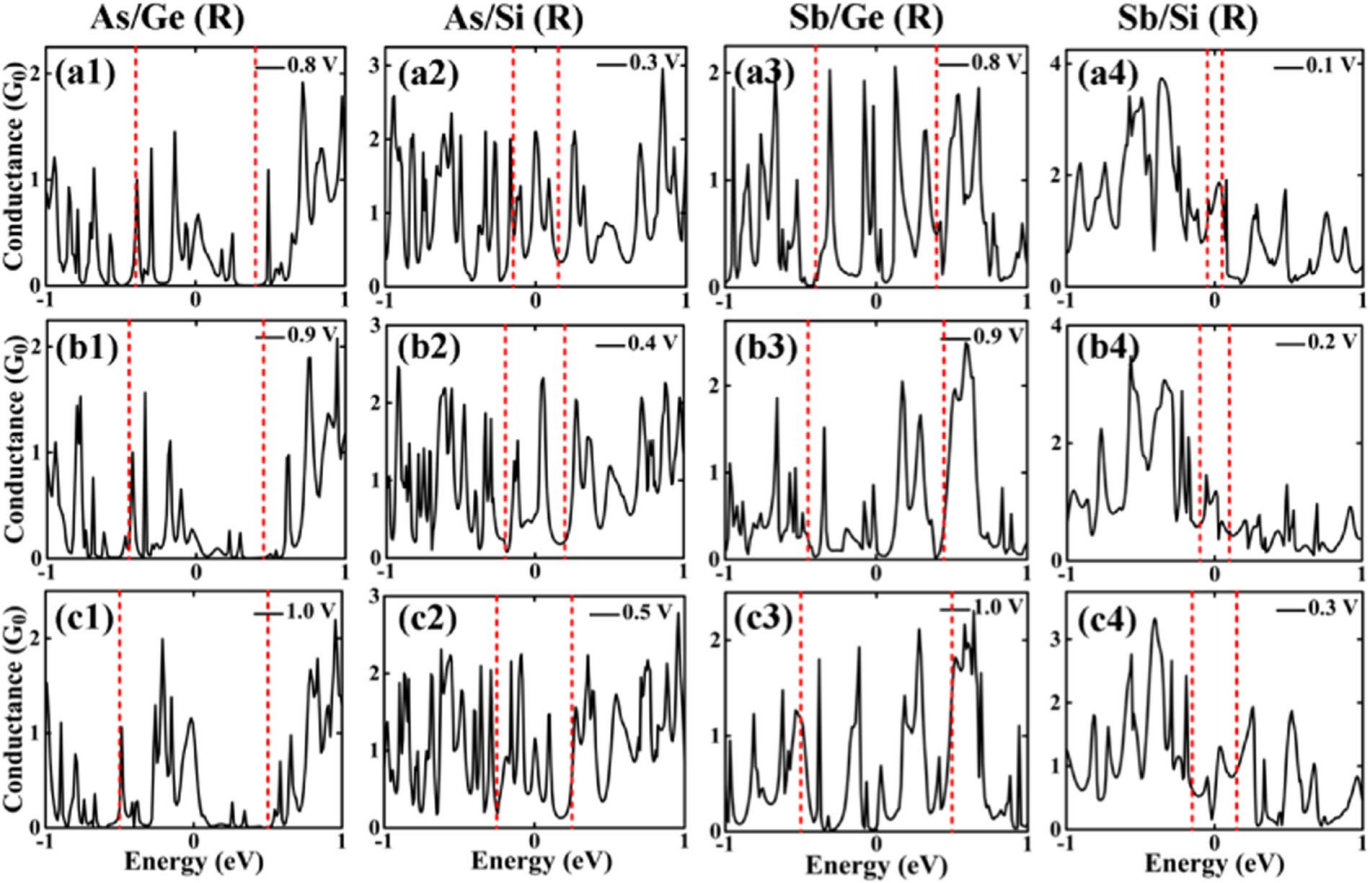
The transmission spectra for three different bias voltages calculated for the considered core/shell nanowires with diameter R.

The As- and Sb- cores wrapped by 1 ML wide Ge and Si shells show a non-linear behavior in their I-V characteristics. The current is calculated to be 13 μA and 25 μA for As/Ge (R) and As/Si (R) NWs, respectively, at V_b_ = 1.0 V. Interestingly, the current is found to decrease at certain values of the applied voltage that leads to a negative differential conductance (NDC). This is an essential property for fast switching in the atomic scale devices. The NDC effect is observed in multiple voltage regions for the As/Ge (R) NW, in the windows 0.5–0.7 V and 0.8–1.0 V. The NDC effect in multiple region of I-V curve has also been observed in AgPt alloyed nanowire in a previous study^58^. The effect is seen in the region 0.3–0.5 V for the As/Si (R) NW as shown in Fig. 5 (inset). The NDC effect is also evident from the electron transmission spectra at the corresponding bias voltages as shown in Fig. 6(a1–c1 and a2–c2). The current (I) through the scattering region has been calculated using Landauer-Buttiker formula that integrates the transmission probability function within the bias window. It is convenient to represent conductance through the scattering region in terms of number of transmission peaks with Van Hove singularities^13^. Greater the number of transmission peaks with Van Hove singularities, larger the current flowing through scattering region at given bias. Therefore, change in the number of transmission peaks within bias window leads to change in current flowing through scattering region. The transmission peaks in these bias windows are observed to decrease and hence the current decreases. For example, in the case of the As/Ge (R) NW, there are 7 transmission peaks in the bias window at 0.8 V which decrease to only 6 peaks at 0.9 V. The number of peaks increases again to 9 peaks in the bias window near V_b_ = 1.0 V. A similar trend is also observed for As/Si (R), Sb/Ge (R) and Sb/Si (R) NWs. The NDC effect for Sb/Ge and Sb/Si is observed at 0.8–1.0 V and 0.1–0.3 V, respectively. The transmission graphs for the Sb-core NWs are presented in Fig. 6 (a3–c3 and a4–c4).

It is noted that the change in current with applied voltage in NDC region is in μA that is large enough to cause the NDC region to persist in the presence of thermal broadening. The NDC effect in a particular region can be attributed to the minor number of conduction channels available for transmission through the scattering region which can be seen as number of transmission peaks with Van Hove singularities available in the bias window at a given bias voltage as shown in Fig. 6.

Further, it is observed that the conductivity of the core-shell NWs abruptly increases, when the core is wrapped by a 2 ML wide shell. The cores wrapped by Si-shells show an ohmic behavior with the highest magnitude of current among the studied NWs. The conductance graphs at different applied voltages are shown in Fig S6 (ESI). It can be seen that a larger number of transmission peaks enter into the bias window at higher applied voltages, which results into an increased current in the corresponding I-V curve. The As- and Sb-cores wrapped by 2 ML Sb-shell show an increment of the current of around 200–300% at V_b_ = 1.0 V as compared to the 1 ML wrapped NWs. The value of the current in the As/Si and Sb/Si NWs also increases to 164 μA and 150 μA, respectively, at 1.0 applied voltages. The I-V curves of the As/Si (2 R), Sb/Ge (2 R) and Sb/Si (2 R) NWs show a linear I-V characteristics that leads to an ohmic behavior of these NWs.

In order to use these NWs as electronic nano-connectors, it is important to investigate their resistance and resistivity. We have calculated the resistance of the core-shell NWs using the formula $V=IR$_,_ where R is the resistance of the NW, and linearly fitting the I-V graphs. Further we have calculated the resistivity from the formula

2 $$\rho =R\frac{A}{l}$$

where, ρ is the resistivity of the NW, A is the cross-sectional area, and *l* is the length of the nanowire. In our case the length of the nanowire is the length of the scattering region.

The calculated values of the resistance and the resistivity are listed in Table 2. The resistivity of the NWs decreases with the increasing of the diameter. Note, that an Au nanowire of diameter 9 nm shows a resistivity of 26.14 μΩ cm, while Cu nanowire of diameter 40 nm shows resistivity of 29.1 μΩ cm. The calculated resistivity in our case is very high. It is important however to mention here that the diameters of the considered NWs are very small (∼1.1 nm and ∼2.0 nm) as compared to the Au (9 nm) and Cu (40 nm) NWs reported in the literature^59–61^.

**Table 2:** Table for resistance and resistivity values corresponding to the core/shell nanowires.

Nanowire (NW)	Nature	Resistance (kΩ)	Resistivity (μΩ cm)	Decrement of resistivity with respect to diameter (%)
AsGe (R)	Semiconductor	71.02	6876.06	
AsSi (R)	Semiconductor	31.25	3246.26	
SbGe (R)	Metallic	48.20	4243.89	
SbSi (R)	Metallic	31.22	2626.46	
AsGe (2 R)	Metallic	20.88	5970.56	13.16
AsSi (2 R)	Metallic	5.02	1156.68	64.36
SbGe (2 R)	Metallic	11.79	2915.03	31.31
SbSi (2 R)	Metallic	5.88	1344.14	48.82
Au^61^	Metallic	1.85	26.14	
NiSi^26^	Metallic	—	9.5	
Cu^59,60^	Metallic	—	29.1	
W^64^	Metallic	—	9–15	
Si^65^	Semiconductor	—	(1–3) × 10^6^	

The nanowires with diameter 1.1 nm show a high resistivity, however we have observed a stiff drop of resistivity as the nanowire diameter increases to 2.0 nm. The decreasing resistivity with increasing nanowire diameter is explained by Fuchs–Sondheimer (FS) theory. As the size of our considered core-shell nanowires are shorter than the mean free path of the electron, therefore, the surface scattering influences the resistance of a nanowire^62,63^ which leads to the decrease in resistivity.

## Conclusions

In summary, we have calculated the electronic and transport properties of novel core/shell nanowire systems. We have considered two types of NWs consisting of As- and Sb-cores. These cores are wrapped by 1 monolayer and 2 monolayers of Ge and Si. All these NWs are fully optimized by density functional theory calculations. We have found that the semiconducting behavior in As/Ge (R) and As/Si (R) and metallic behavior in Sb/Ge (R) and Sb/Si (R) are due to the intrinsic strain experienced by the core region. Both the As and Sb cores, wrapped by 1 ML of Ge- and Si-shell show a non-linear behavior and a negative differential conductance which may be useful for the realization of atomic scale devices The current in the given NWs structures has a strong dependence on the diameter of NWs. The core/shell nanowires with higher diameters show high current values in accordance to the applied voltage. The As/Si (2 R), Sb/Ge (2 R) and Sb/Si (2 R) NWs show an ohmic behavior. The transition from a semiconducting to a metallic behaviour in As/ Si and As/Ge NWs with the change in the diameter has been found. The resistivity of the considered NWs decreases with the increasing of the diameters. These core-shell nanowires with tunable transport properties and ohmic behaviour show a potential for applications as electron connectors in nanoelectronic devices.

## Electronic supplementary material

Electronic supplementary information (ESI)
