# Supplementary material for: Si and Ge based metallic core/shell nanowires for nano-electronic device applications
Source: Sci Rep. 2018 Nov 15;8:16885. doi: 10.1038/s41598-018-35225-6 (PMC6237903; doi:10.1038/s41598-018-35225-6)
Supplement: Supplementary file 1 — Electronic supplementary information (ESI) [file 41598_2018_35225_MOESM1_ESM.docx]

**Electronic supplementary information (ESI)**

**Si and Ge based metallic core/shell nanowires for nano-electronic device applications**

Prabal Dev Bhuyan^1, 4^_,_ Ashok Kumar^2^, Yogesh Sonvane^3^, P. N. Gajjar^4^,

Rita Magri^5^ and Sanjeev K. Gupta^1,*^

^1^Computational Materials and Nanoscience Group, Department of Physics and Electronics,

St. Xavier's College, Ahmedabad 380009, India

^2^Department of Physical Sciences, School of Basic and Applied Sciences, Central University of Punjab, Bathinda, Punjab 151001, India.

^3^Department of Applied Physics, S.V. National Institute of Technology, Surat 395007, India

^4^Department of Physics, Gujarat University, Ahmedabad 380009, India

^5^ Department of Physics, Informatics and Mathematics (FIM), University of Modena and Reggio Emilia, Via Campi 213/A, Modena, Italy


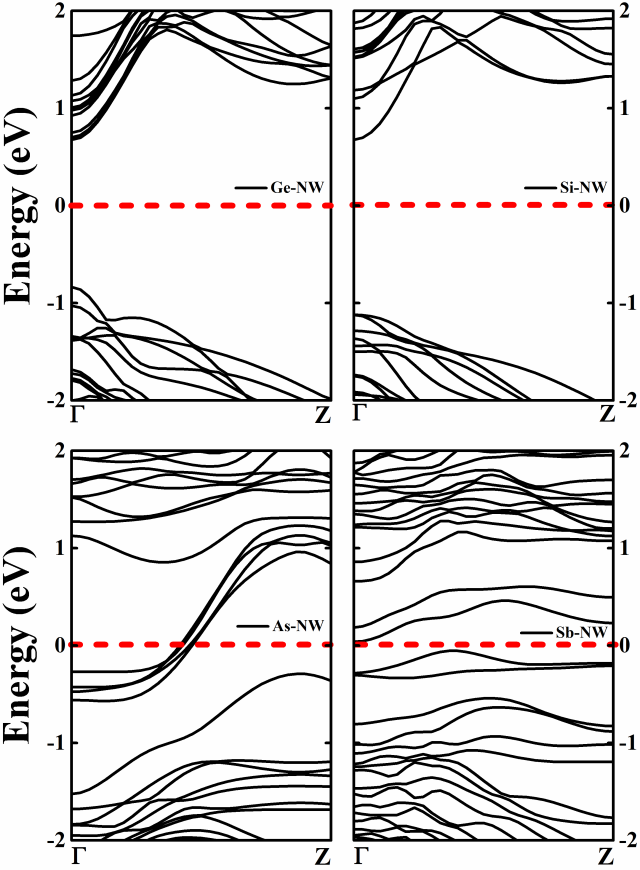


**Figure S1:** The calculated electronic band structure of pristine NWs: Ge-, Si-, As- and Sb-NW.


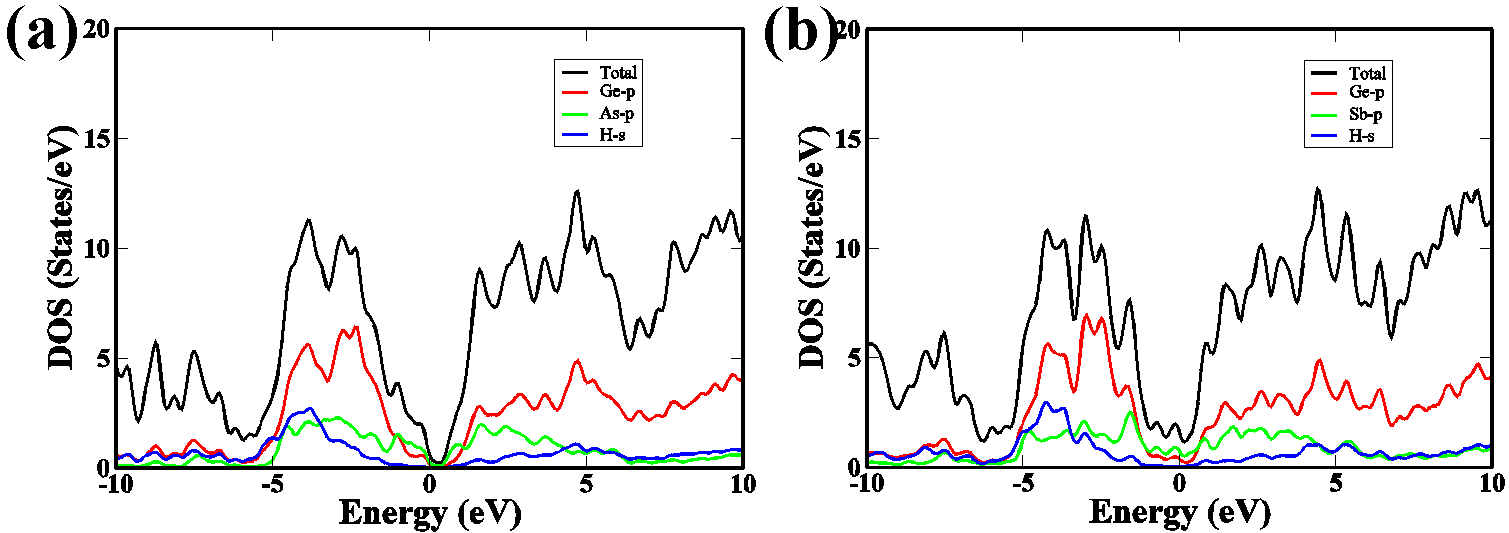

**Figure S2:** The calculated partial density of states (PDOS) for As/Ge and Sb/Ge core/shell NW. The diameter of the NW is 12Å, while core is of 5Å.


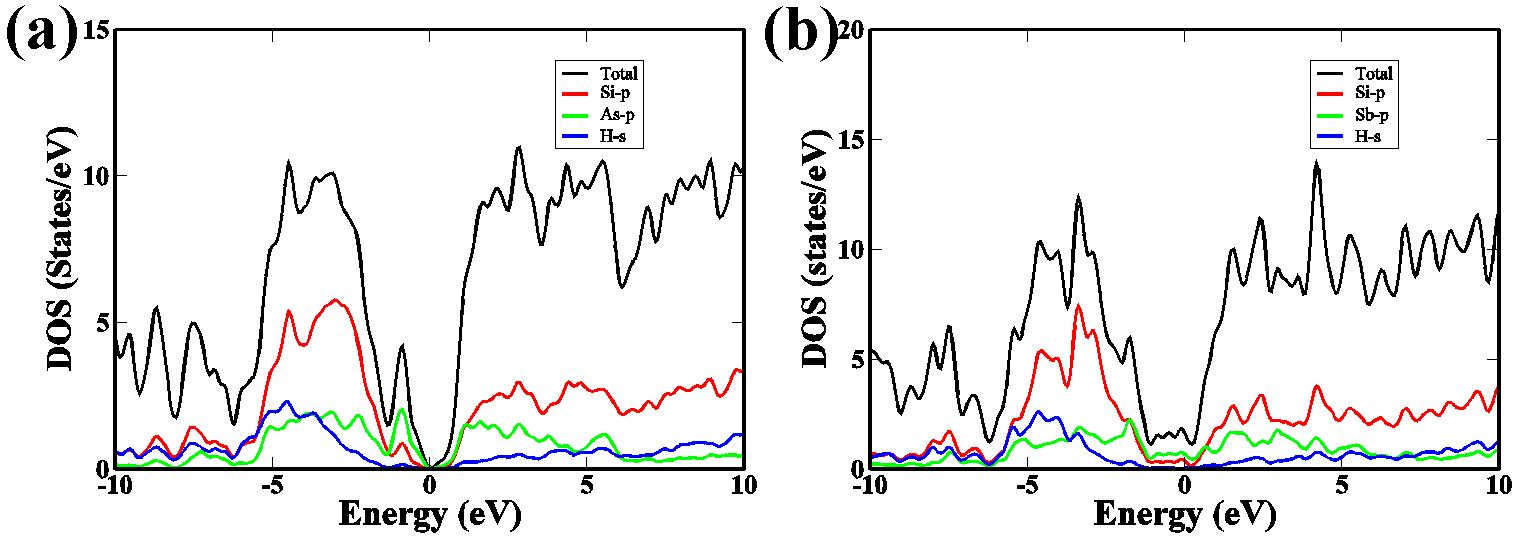

**Figure S3:** The calculated partial density of states (PDOS) for As/Si and Sb/Si core/shell NW. The diameter of the NW is 12Å, while core is of 5Å.


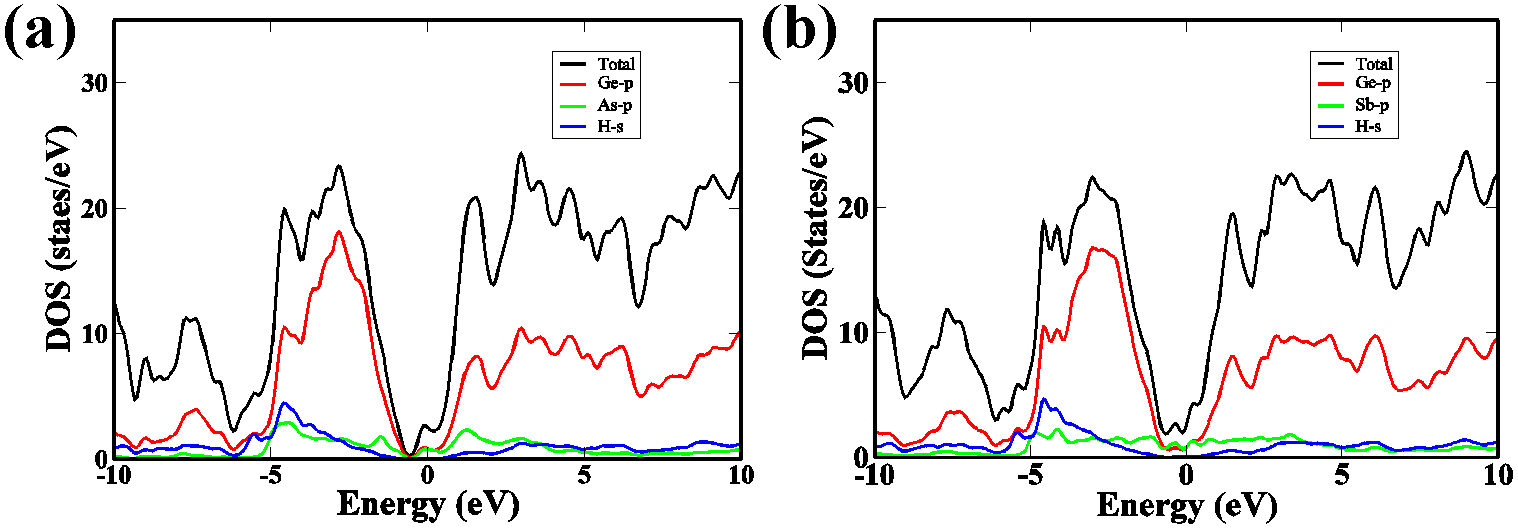

**Figure S4:** The calculated partial density of states (PDOS) for As/Ge and Sb/Ge core/shell NW. The diameter of the NW is 20Å, while core is of 5Å.


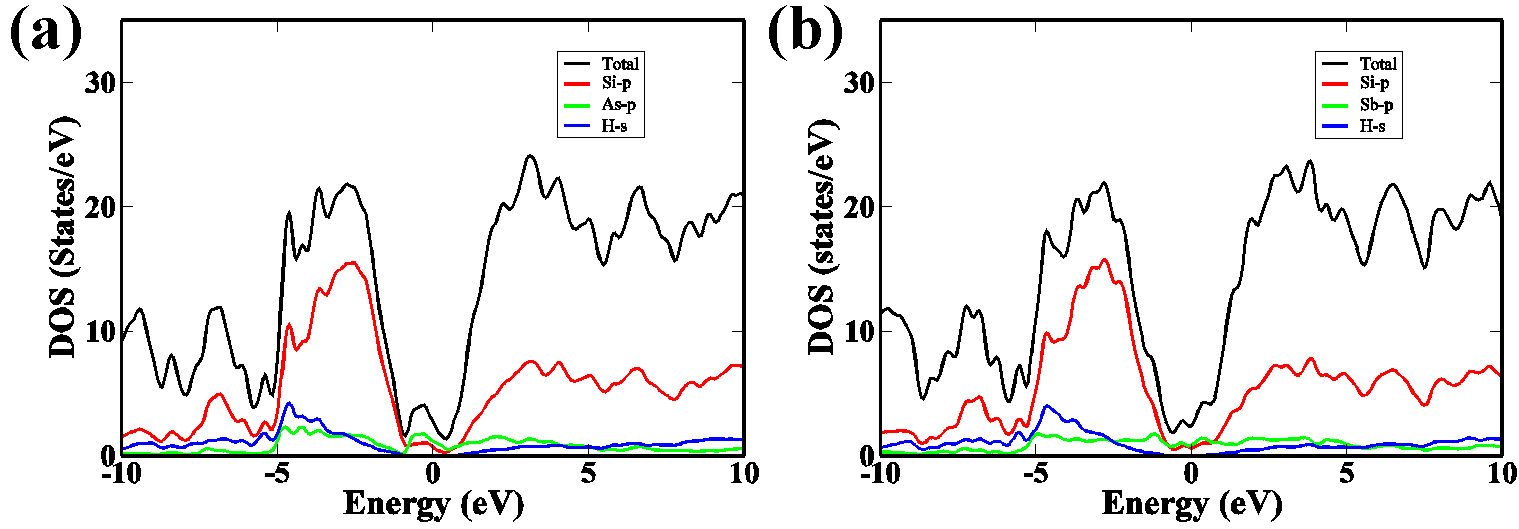

**Figure S5:** The calculated partial density of states (PDOS) for As/Si and Sb/Si core/shell NW. The diameter of the NW is 20Å, while core is of 5Å.


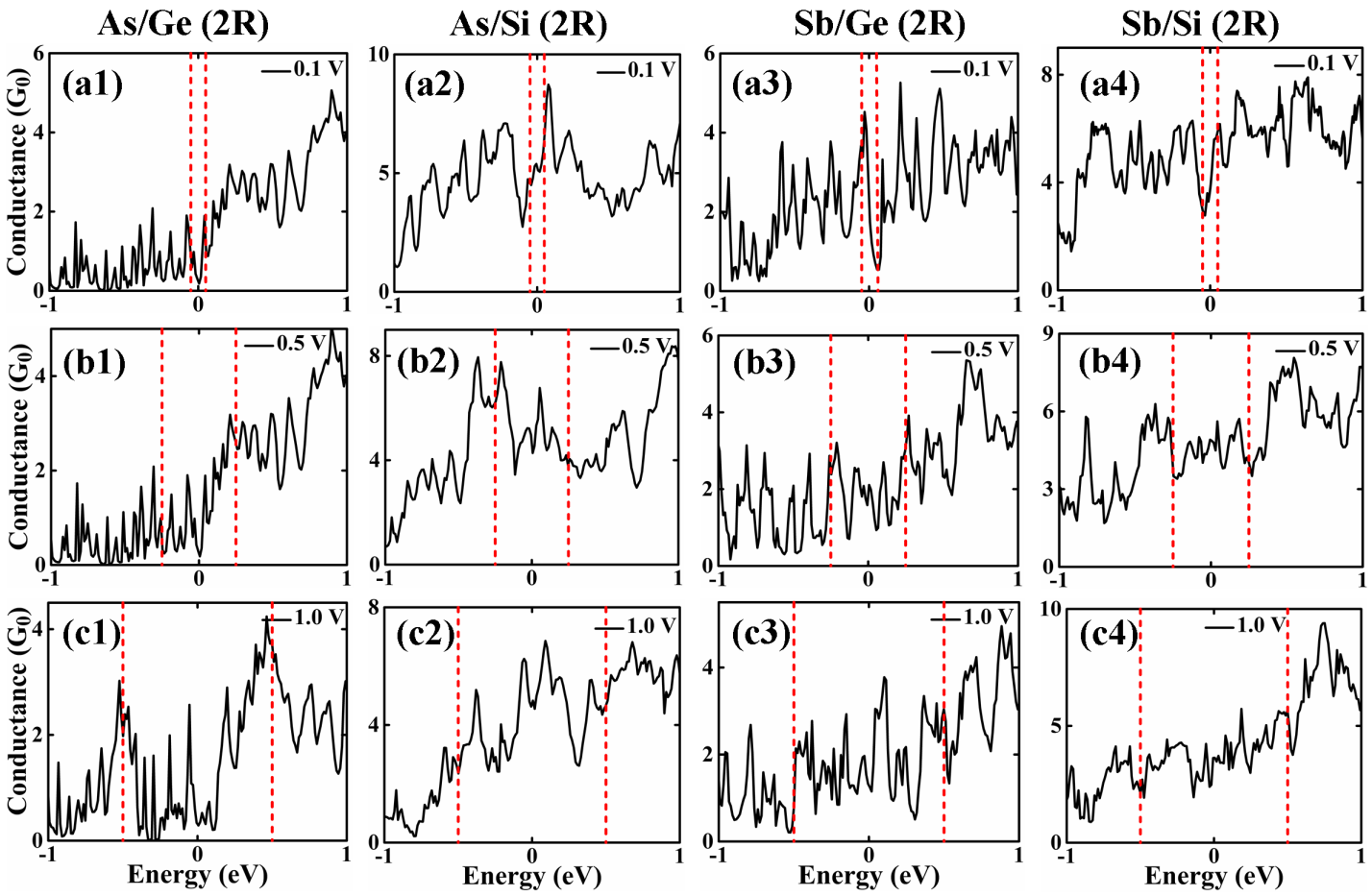

**Figure S6:** The transmission spectrums for three different bias voltage calculated for the considered core/shell nanowires.


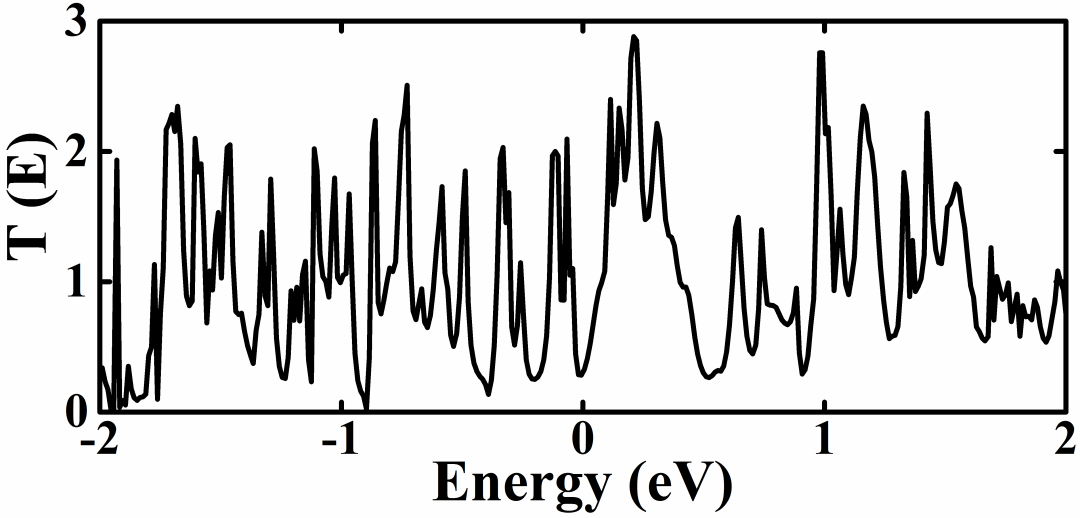


**Figure S7:** The transmission spectrum for As/Si NW at zero bias voltage. The length of the central scattering region is around 116Å, consisted of four primitive cells.
